# Genome Anatomy of *Streptococcus parasanguinis* Strain C1A, Isolated from a Patient with Acute Exacerbation of Chronic Obstructive Pulmonary Disease, Reveals Unusual Genomic Features

**DOI:** 10.1128/genomeA.00541-15

**Published:** 2015-05-28

**Authors:** Kok-Gan Chan, Kim Tien Ng, Yong Kek Pang, Teik Min Chong, Adeeba Kamarulzaman, Wai-Fong Yin, Kok Keng Tee

**Affiliations:** a Division of Genetics and Molecular Biology, Institute of Biological Sciences, Faculty of Science, University of Malaya, Kuala Lumpur, Malaysia; b Faculty of Medicine, University of Malaya, Kuala Lumpur, Malaysia

## Abstract

*Streptococcus parasanguinis* causes invasive diseases. However, the mechanism by which it causes disease remains unclear. Here, we describe the complete genome sequence of *S. parasanguinis* C1A, isolated from a patient diagnosed with an acute exacerbation of chronic obstructive pulmonary disease. Several genes that might be associated with pathogenesis are also described.

## GENOME ANNOUNCEMENT

Viridans streptococci are a group of Gram-positive bacteria that have been reported to cause infective endocarditis (1). The mechanisms by which viridans streptococci, specifically *Streptococcus parasanguinis*, cause infections remain unclear. Here, we describe the genome sequence of *S. parasanguinis*, isolated from an individual diagnosed with an acute exacerbation of chronic obstructive pulmonary disease. We also investigated several putative virulence factors that might be associated with the pathogenesis.

The bacteria were isolated from a sputum sample from a consenting subject and were then characterized by Microflex matrix-assisted laser desorption ionization–time of flight mass spectrometry (MALDI-TOF MS) (Bruker Daltonik GmbH, Leipzig, Germany) equipped with the flexControl and Bruker MALDI Biotyper real-time classification softwares (2). The genomic DNA of the bacteria was extracted using the MasterPure Gram-positive DNA purification kit (Epicentre, Madison, WI), according to the manufacturer’s protocol. Whole-genome sequencing was performed using an Illumina MiSeq sequencer (Illumina, Inc., CA). The generated reads were trimmed and assembled *de novo* using CLC Genomics Workbench 6.0 (CLC bio, Denmark) (3) and annotated by Rapid Annotations using Subsystems Technology (RAST) (4). Targeted sequences were investigated using the NCBI Basic Local Alignment Search Tool, MEROPS peptidase, and InterPro Databases (5–7).

A total of 79 contigs with an average coverage of 68.7× were generated. The *N*_50_ and G+C content of the draft genome are 39,293 bp and 42.0%, respectively. RAST analysis indicated that the closest neighbor of *S. parasanguinis* strain C1A is *S. parasanguinis* ATCC 15912. Besides a fibronectin/fibrinogen binding gene, a collagen-binding surface protein-encoding gene was identified. This provides insight into the possible mechanism of adherence to the host cell. Another adherence tool, namely, adhesin protein, was observed, highlighting the wide spectrum of adhesins used by *S. parasanguinis* (8). An oligopeptide-binding protein SarA-encoding gene, which is important for colonization, was also identified (9).

Other virulence factors, such as genes encoding serine protease, which has been implicated in the pathogenesis of various infections (10, 11), were discovered. The InterPro and MEROPS databases suggest this cell wall-associated S8A serine protease carries a peptidase S8 domain (PF00082) and a catalytic triad in the order aspartic acid, histidine, and serine in the sequence is likely to be involved in pathogenesis (5). Also, it carries a bacterial immunoglobulin/albumin-binding domain (IPR009063) and an extracellular matrix-binding protein domain, Ebh (IPR011490), near the C terminus. The enolase gene, which plays a role in catalyzing the reversible conversion of 2-phosphoglycerate into phosphoenolpyruvate, was found in the genome. It has been reported to bind to plasminogen, potentially facilitate the bacterium in surface-associated proteolytic activity, and contribute to the degradation of the extracellular matrix (12, 13). In addition, antibiotic resistance-related gene products were discovered, namely, tetracycline resistance protein TetM, multidrug transporter, and aminoglycoside phosphotransferase. The elimination of this bacterium might be challenging due to the presence of antibiotic resistance genes. Thus, the drug regime used in the treatment of viridans streptococci-related infection might be a major challenge.

### Nucleotide sequence accession number. 

The genome sequence of *S. parasanguinis* C1A has been deposited in GenBank under the accession no. JMRV00000000. The version described in this paper is the first version.
